# Supplementary material for: Outside-in engineering of cadherin endocytosis using a conformation strengthening antibody
Source: Nat Commun. 2025 Jan 29;16:1157. doi: 10.1038/s41467-025-56478-6 (PMC11779849; doi:10.1038/s41467-025-56478-6)
Supplement: Supplementary file 1 — Supplementary Information [file 41467_2025_56478_MOESM1_ESM.pdf]

Supplementary Information

Title: Outside-in engineering of cadherin endocytosis using a conformation strengthening antibody.

Authors: Bin Xie [1, ¥], Shipeng Xu [2, ¥], Sanjeevi Sivasankar [1, 2, *]

Affiliations: [1] Biophysics Graduate Group, University of California, Davis, CA; [2] Department of Biomedical Engineering, University of California, Davis, CA.

[¥]Equal Contribution

***Corresponding Author:** ssivasankar@ucdavis.edu

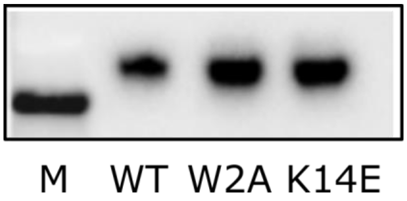

Supplementary Figure 1. Western blots confirm that CQY684 recognizes WT, W2A, and K14E Pcad. The molecular weight marker (M) corresponds to a molecular weight of 75 kD.

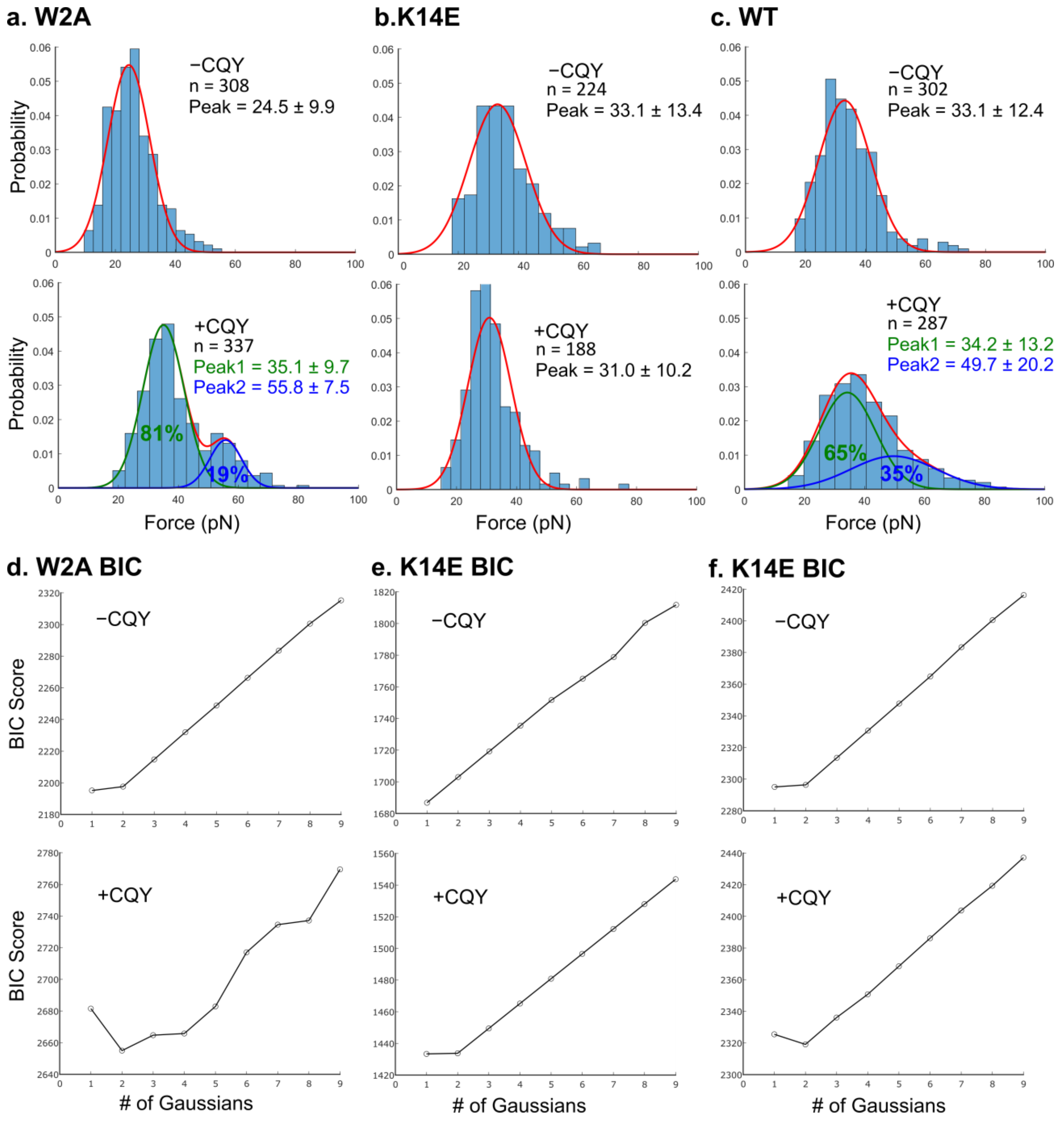

Supplementary Figure 2. Histograms of unbinding forces with or without CQY684 for W2A, K14E, and WT Pcad, along with the corresponding Bayesian Information Criterion (BIC) test. Histograms of unbinding forces with or without CQY684 Fab for the experiments using (a) W2A, (b) K14E, and (c) WT Pcad. (d) (e) (f) Corresponding BIC testing shows that all force distributions are best described by a single Gaussian distribution expect the W2A + CQY and WT + CQY conditions, which are best described by two Gaussian distributions.

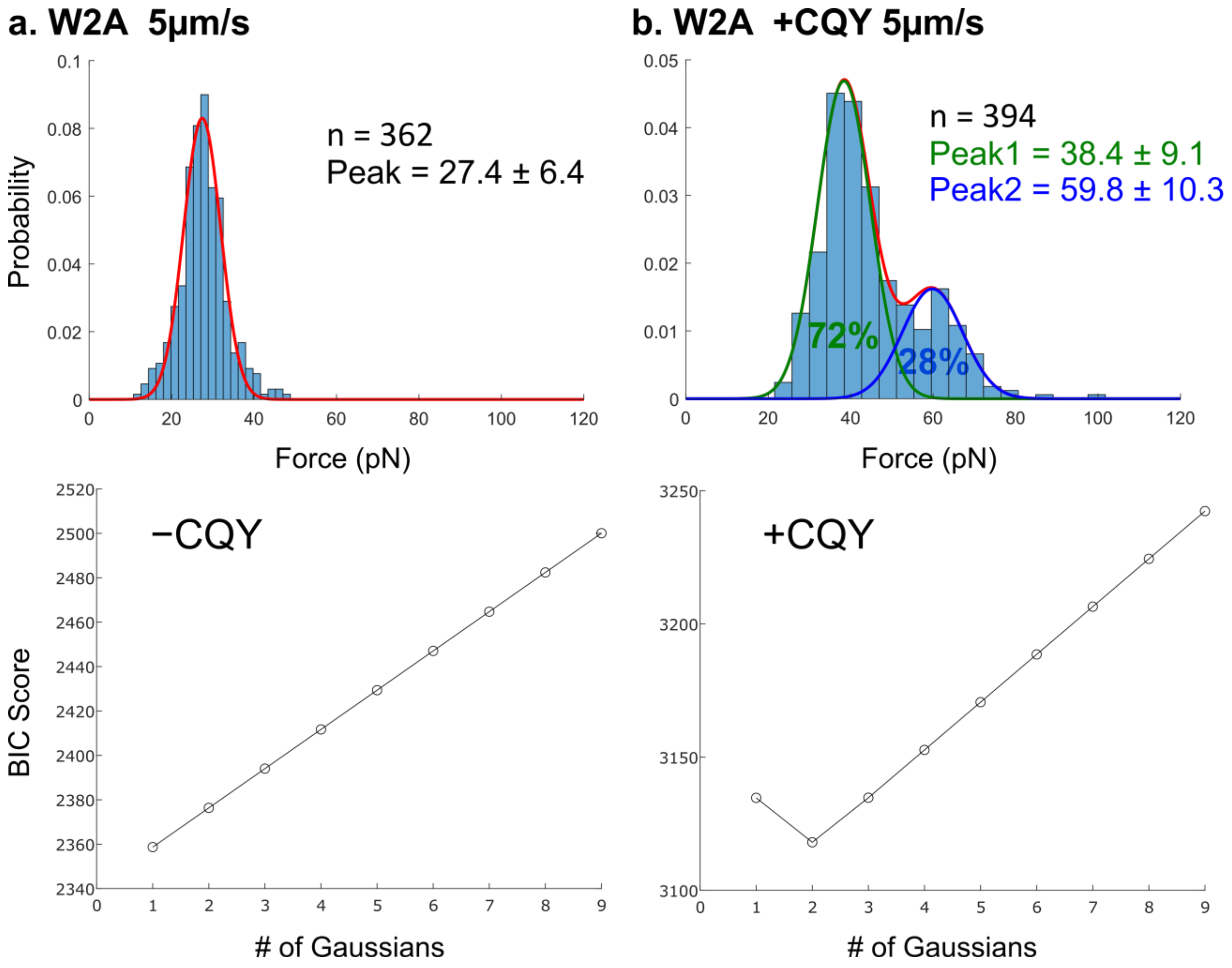

Supplementary Figure 3. AFM experiments with Pcad W2A performed at a higher pulling velocity and the corresponding Bayesian Information Criterion (BIC) test. Histograms and BIC tests of unbinding forces (a) without or (b) with CQY684 Fab. These experiments were done using a higher pulling velocity of 5μm/s. Single Gaussian distribution in the -CQY condition, and bimodal Gaussian distribution in the +CQY conditions were measured.

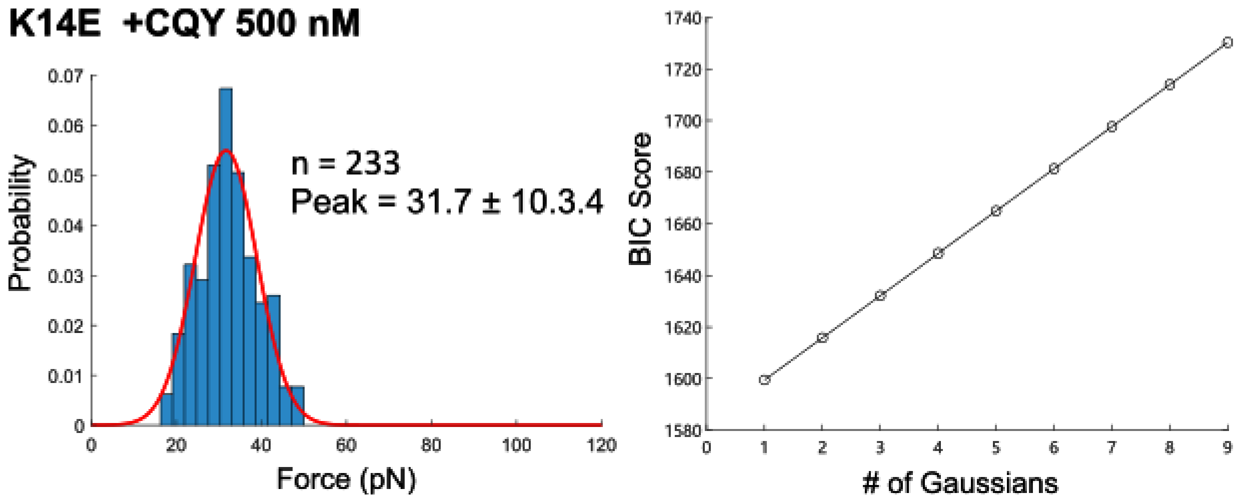

Supplementary Figure 4. AFM experiments with the Pcad K14E mutant, performed at higher CQY684 concentration and the corresponding Bayesian Information Criterion (BIC) test. The unbinding force histograms in the presence of 500 nM CQY684 Fab are best described by a single Gaussian distribution.

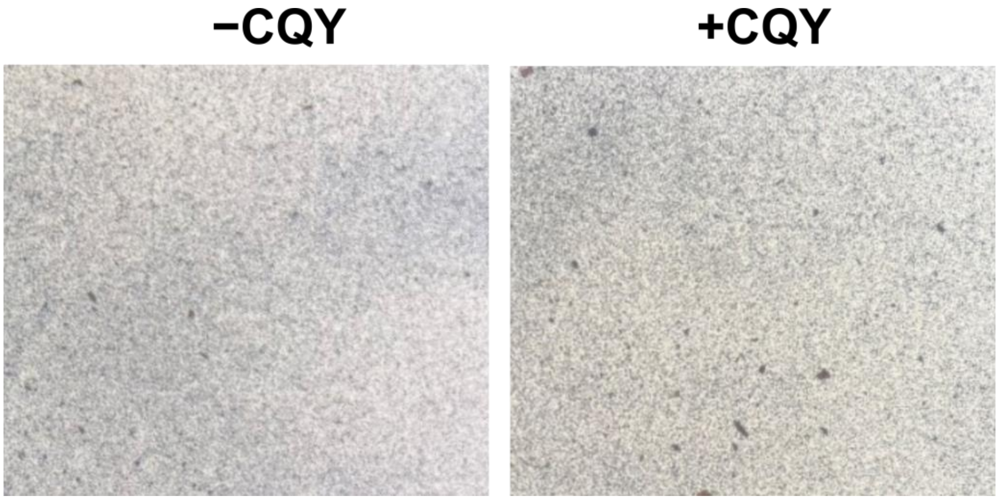

Supplementary Figure 5. Addition of CQY684 does not alter K14E Pcad bead aggregation. Significant bead aggregation was not observed for K14E Pcad with or without CQY684.

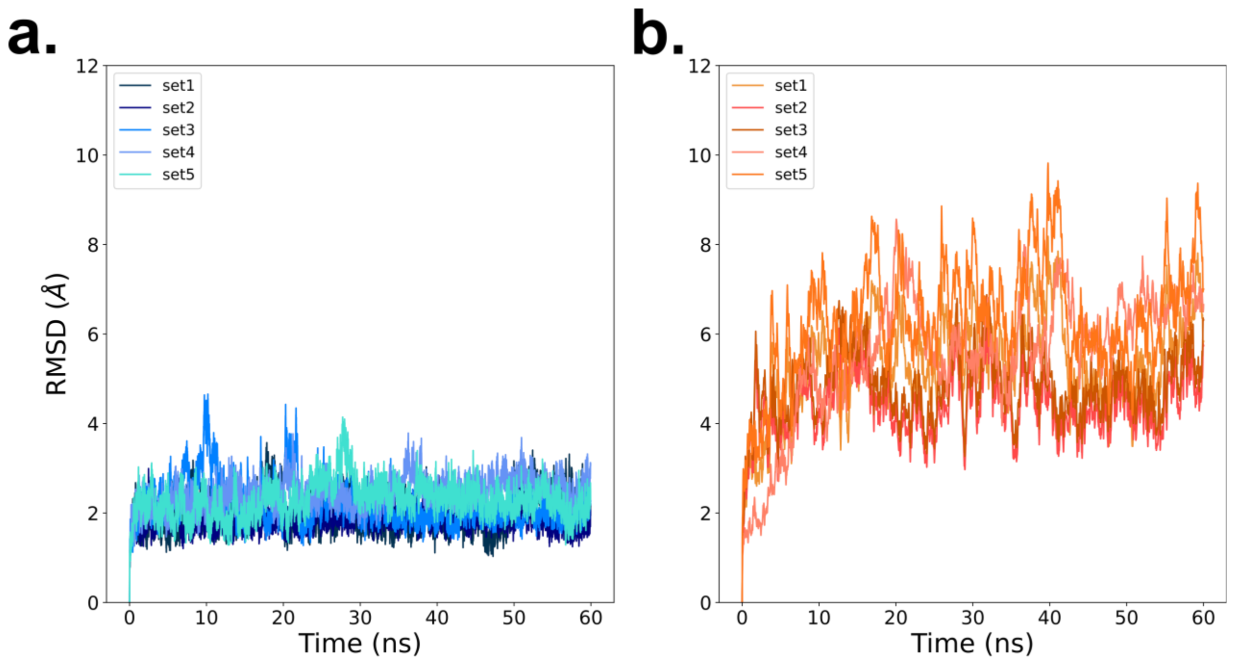

Supplementary Figure 6. Protein backbone RMSD in MD simulations relative to the initial structures at the start of simulation. RMSD values measured for each set in the two conditions (a) −CQY condition, and (b) +CQY condition. Stabilization of RMSD values suggest that structures are well equilibrated.

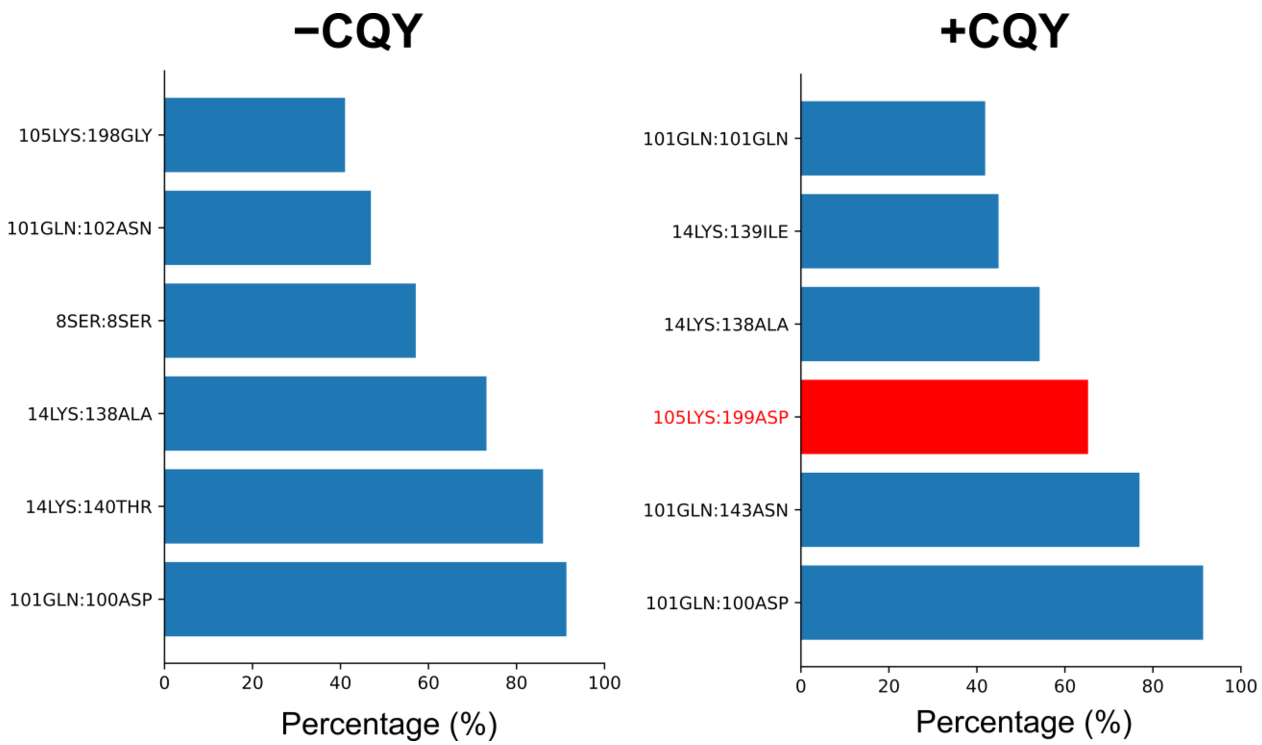

Supplementary Figure 7. Electrostatic interactions between Pcads in an X-dimer change upon interaction with CQY684. Bar plots of all the electrostatic interactions which include salt bridges and hydrogen bonds that are persistent over 40% of the total simulation time are shown above. Salt bridge interactions are highlighted in red and hydrogen bonds interaction are colored in blue. The addition of CQY684 introduced a novel salt bridge 105LYS:199ASP.

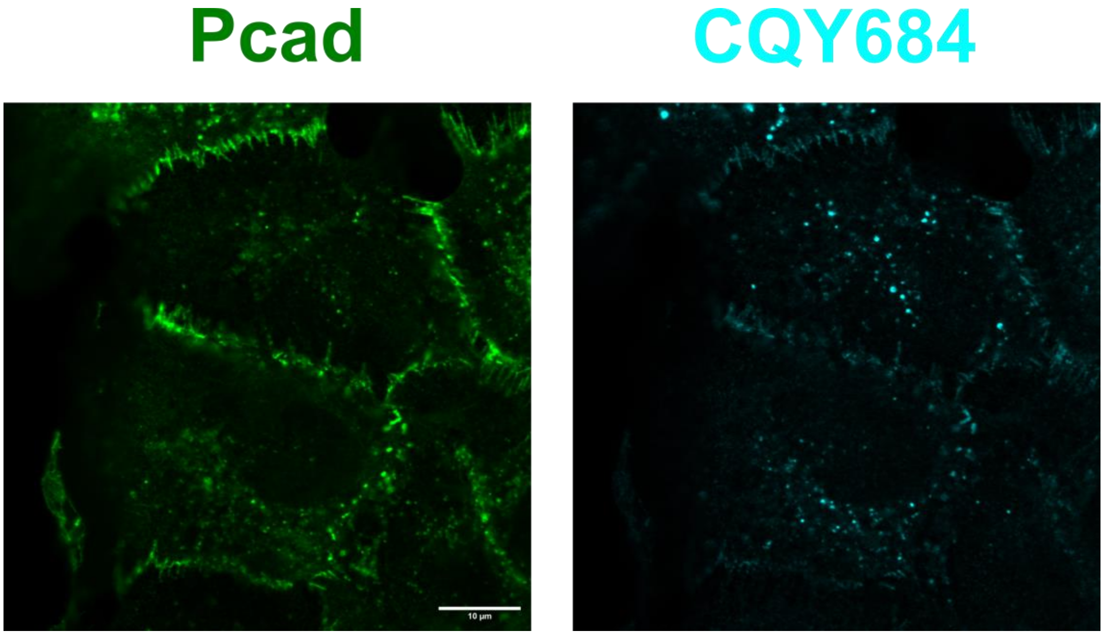

Supplementary Figure 8. CQY684 is localized at the cell surface at the timepoints of cell experiments. Staining of Pcad (left) and CQY684 (right) after the addition of CQY684 and overnight incubation. A fraction of CQY684 remains bound on the cell surface.

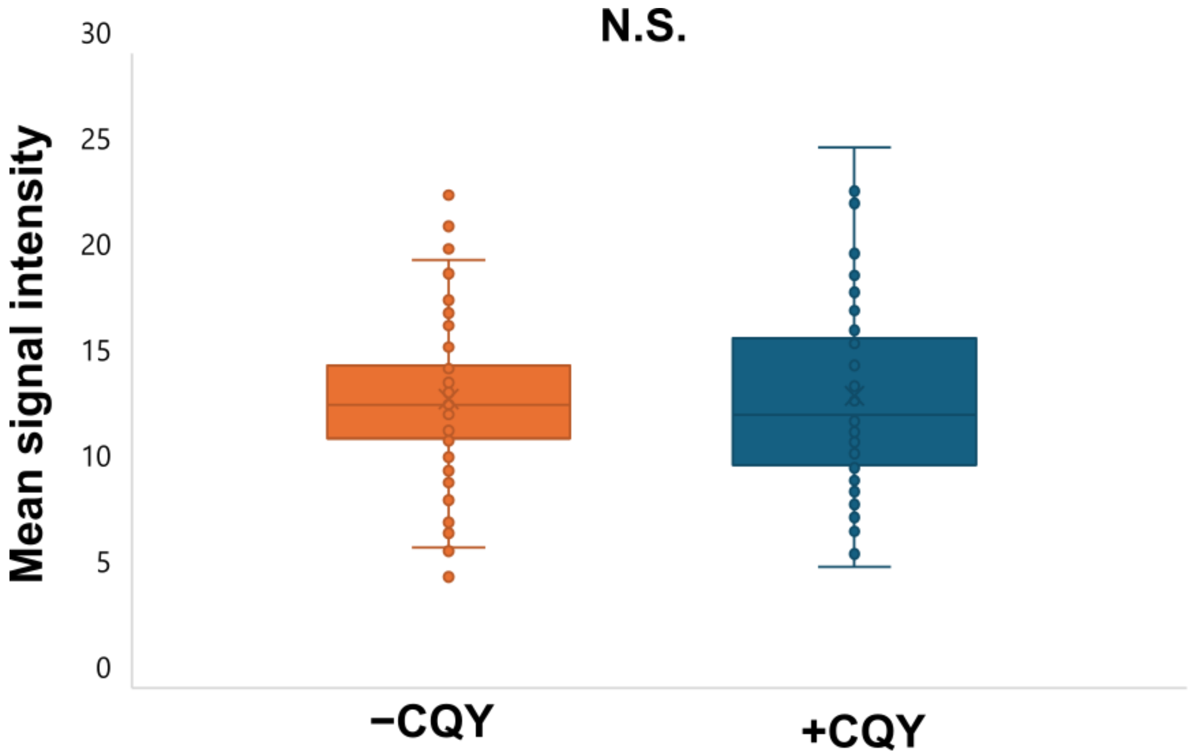

Supplementary Figure 9. Mean LAMP1 signal intensity was similar in cells with or without CQY684 treatment. For the -CQY condition, the average LAMP1 intensity was 13.80 ± 4.45, and for the +CQY condition, the average LAMP1 intensity was 13.67 ± 3.78. The P-value of 0.84 indicates no significant difference between these two conditions**.**

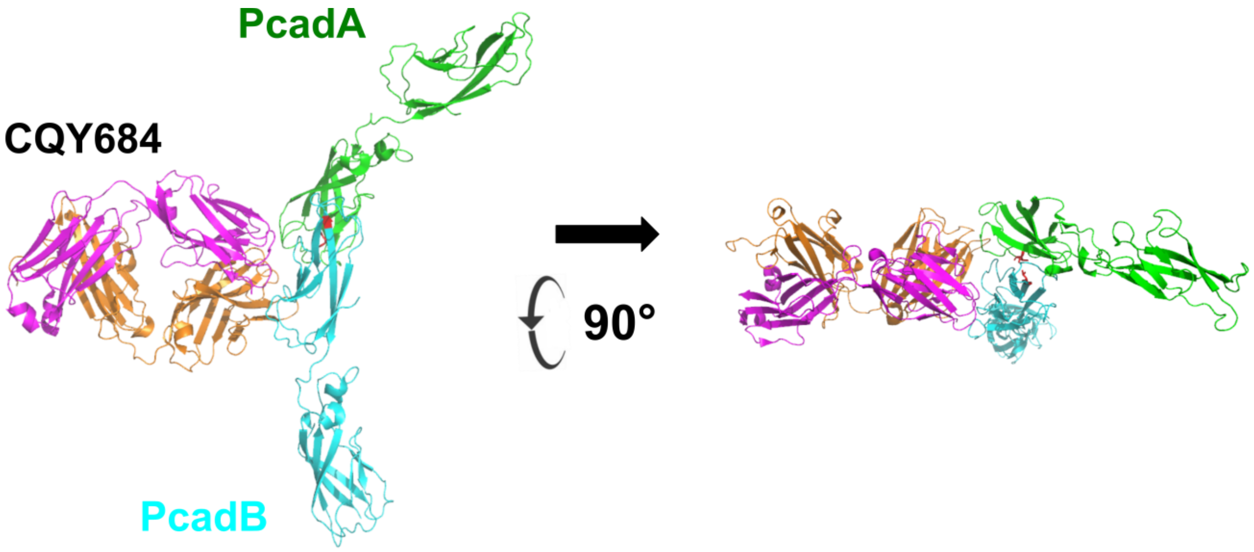

Supplementary Figure 10. CQY684 binding does not obstruct the Pcad *cis* dimer interface. CQY684 was aligned on a Pcad *cis* dimer (colored in green and cyan, PDB code: 4ZMX). The hydrophobic core of the Pcad *cis* interface, I175 and V81 is highlighted in red. No interference is observed between CQY684 and the Pcad *cis* dimer.

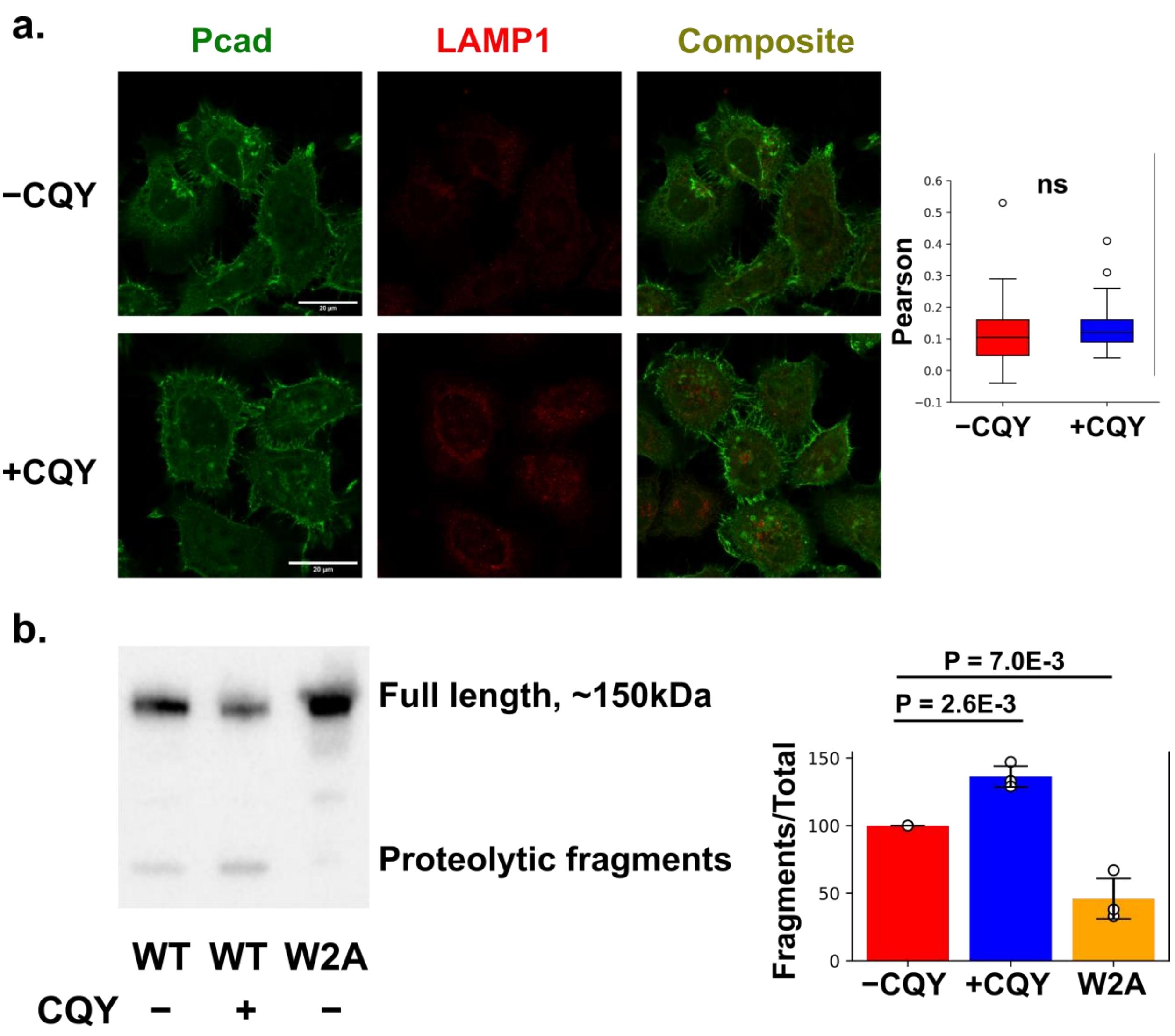

Supplementary Figure 11. CQY684 does not induce W2A Pcad colocalization with LAMP1 due to low proteolysis activity in Pcad W2A cell line. (a) Immunofluorescence confocal imaging shows that W2A-Pcad do not colocalize with the lysosomal marker LAMP1 in the presence or absence of CQY684. N = 50 cells for both conditions. Student t-test was performed, and no significant difference was observed for colocalization Pearson's coefficients. (b) Left panel: western blots detecting Pcad observed two bands in the cell lysate across the three conditions: 'WT -CQY', 'WT +CQY', and 'W2A'. The top band, which has molecular weight ~150kDa, corresponds to the full length Pcad, while the bottom band, which has molecular weight ~75kDa, corresponds to the proteolytic fragments of Pcad. Right panel: Bar-plot of three replicates. All values are compared to the −CQY conditions. The proteolytic fragments of Pcad increase in the +CQY conditions, corresponding to more endo-lysosomal activity induced by the addition of CQY684, but dramatically decrease in the W2A mutation. This suggests that the W2A mutation silences proteolytic activity, which is required for endo-lysosomal trafficking of Pcad.

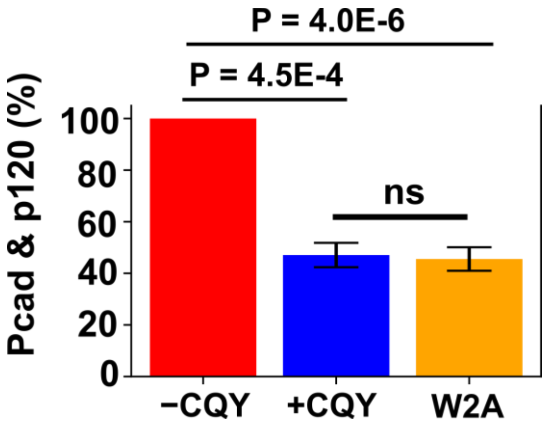

Supplementary Figure 12. The amount of ‘effective Pcad’ is reduced in both +CQY and W2A conditions. Bar-plot of three replicates. All values are compared to the −CQY conditions.

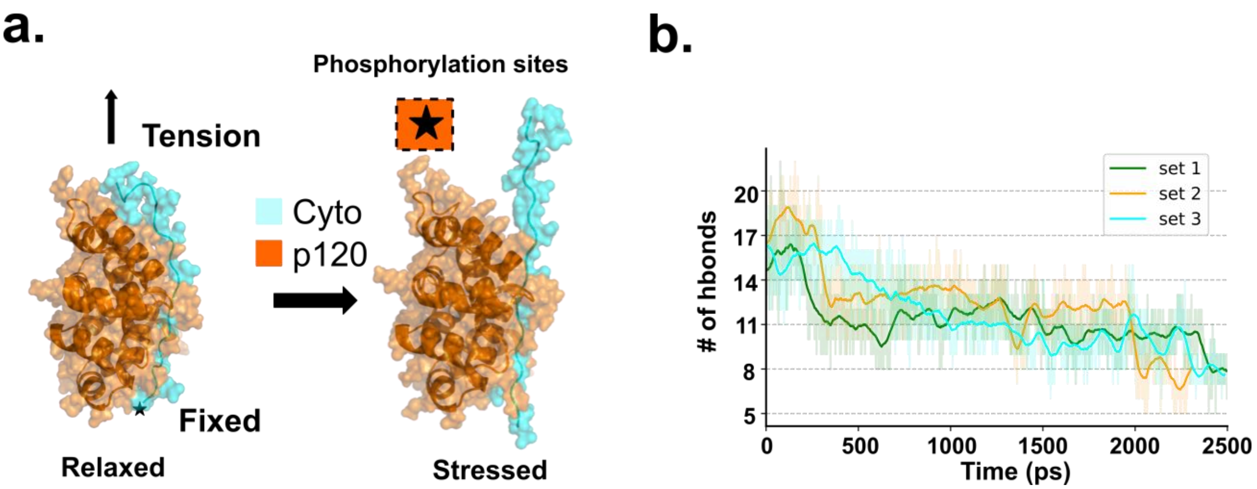

Supplementary Figure 13. Model for how X-dimer formation could induce conformational changes in the cadherin cytoplasmic region and expose p120 phosphorylation sites. A previous model suggests that cell-cell junctions formed by S-dimers are wider than those formed by X-dimers (reference 10 in the manuscript). Consequently, X-dimer formation would generate a tensile stress on the cadherin cytoplasmic region. To test if this force would induce a conformational change in the cadherin cytoplasmic region which would subsequently expose p120 phosphorylation sites, we performed MD and SMD simulations on the crystal structure of the cadherin cytoplasmic juxtamembrane (JMD) core region (residues 758-775) interacting with portion of p120 (residues 359-511). (a) The cadherin cytoplasmic region is in cyan while p120 is in brown. We first used 20 ns MD simulations to equilibrate the structures and observed that the cadherin cytoplasmic tail was tightly bound to p120 (left panel). To mimic a tensile force on the cadherin cytoplasmic tail, we performed SMD simulations where we fixed the C-terminal residue of the cytoplasmic regions and applied a constant stretching force to the N-terminal residue (right panel). (b) Upon application of a tensile force, the hydrogen bond network between p120 and the cadherin cytoplasmic region decreased over time, suggesting that the stress on the cytoplasmic region weakens these interactions, thereby exposing p120 phosphorylation sites.
