# Supplementary material for: Outside-in engineering of cadherin endocytosis using a conformation strengthening antibody
Source: Nat Commun. 2025 Jan 29;16:1157. doi: 10.1038/s41467-025-56478-6 (PMC11779849; doi:10.1038/s41467-025-56478-6)
Supplement: Supplementary file 2 — Description of Additional Supplementary Files [file 41467_2025_56478_MOESM2_ESM.pdf]

Description of Additional Supplementary Files

File Name: Supplementary Movie 1
Description: Example constant-force SMD simulation.
