# Supplementary material for: Outside-in engineering of cadherin endocytosis using a conformation strengthening antibody
Source: Nat Commun. 2025 Jan 29;16:1157. doi: 10.1038/s41467-025-56478-6 (PMC11779849; doi:10.1038/s41467-025-56478-6)
Supplement: Supplementary file 4 — Reporting Summary [file 41467_2025_56478_MOESM4_ESM.pdf]

nature portfolio

Corresponding author(s): Sanjeevi Sivasankar

Last updated by author(s): Sep 18, 2024

Reporting Summary

Nature Portfolio wishes to improve the reproducibility of the work that we publish. This form provides structure for consistency and transparency in reporting. For further information on Nature Portfolio policies, see our Editorial Policies and the Editorial Policy Checklist.

Statistics

For all statistical analyses, confirm that the following items are present in the figure legend, table legend, main text, or Methods section.

n/a	Confirmed	
☐	☒	The exact sample size (*n*) for each experimental group/condition, given as a discrete number and unit of measurement
☐	☒	A statement on whether measurements were taken from distinct samples or whether the same sample was measured repeatedly
☐	☒	The statistical test(s) used AND whether they are one- or two-sided<br>*Only common tests should be described solely by name; describe more complex techniques in the Methods section.*
☒	☐	A description of all covariates tested
☐	☒	A description of any assumptions or corrections, such as tests of normality and adjustment for multiple comparisons
☐	☒	A full description of the statistical parameters including central tendency (e.g. means) or other basic estimates (e.g. regression coefficient) AND variation (e.g. standard deviation) or associated estimates of uncertainty (e.g. confidence intervals)
☐	☒	For null hypothesis testing, the test statistic (e.g. *F*, *t*, *r*) with confidence intervals, effect sizes, degrees of freedom and *P* value noted<br>*Give P values as exact values whenever suitable.*
☐	☒	For Bayesian analysis, information on the choice of priors and Markov chain Monte Carlo settings
☒	☐	For hierarchical and complex designs, identification of the appropriate level for tests and full reporting of outcomes
☐	☒	Estimates of effect sizes (e.g. Cohen's *d*, Pearson's *r*), indicating how they were calculated

Our web collection on statistics for biologists contains articles on many of the points above.

Software and code

Policy information about availability of computer code

Data collection	AFM data was acquired using PicoView 1.20 software from Keysight Technologies. Python 2.7 was used to automate AFM operation. Western blots data was acquired using Image Lab from Bio-Rad. Molecular Dynamics simulations were performed using Gromacs 2020.1. All imaging data was acquired using Leica Stellaris confocal microscope platform.
Data analysis	AFM analysis was performed with custom code using MATLAB R2022B. Molecular dynamics simulations were visualized with VMD 1.9.3, figures were created with PyMOL open-source 1.8.4.0, and RMSDs were calculated using Gromacs 2020.2. Imaging data was analyzed using Excel 2020 (Microsoft), ImageJ, and Python3.

For manuscripts utilizing custom algorithms or software that are central to the research but not yet described in published literature, software must be made available to editors and reviewers. We strongly encourage code deposition in a community repository (e.g. GitHub). See the Nature Portfolio guidelines for submitting code & software for further information.

Data

Policy information about availability of data

All manuscripts must include a data availability statement. This statement should provide the following information, where applicable:

- Accession codes, unique identifiers, or web links for publicly available datasets
- A description of any restrictions on data availability
- For clinical datasets or third party data, please ensure that the statement adheres to our policy

All data produced by this research have been made available in the manuscript and supporting information. Simulation input files and a coordinate file of the final

output have been deposited in a public repository (https://doi.org/10.5281/zenodo.13787595)

Research involving human participants, their data, or biological material

Policy information about studies with human participants or human data. See also policy information about sex, gender (identity/presentation), and sexual orientation and race, ethnicity and racism.

Reporting on sex and gender	*Use the terms sex (biological attribute) and gender (shaped by social and cultural circumstances) carefully in order to avoid confusing both terms. Indicate if findings apply to only one sex or gender; describe whether sex and gender were considered in study design; whether sex and/or gender was determined based on self-reporting or assigned and methods used.*<br>*Provide in the source data disaggregated sex and gender data, where this information has been collected, and if consent has been obtained for sharing of individual-level data; provide overall numbers in this Reporting Summary. Please state if this information has not been collected.*<br>*Report sex- and gender-based analyses where performed, justify reasons for lack of sex- and gender-based analysis.*
Reporting on race, ethnicity, or other socially relevant groupings	*Please specify the socially constructed or socially relevant categorization variable(s) used in your manuscript and explain why they were used. Please note that such variables should not be used as proxies for other socially constructed/relevant variables (for example, race or ethnicity should not be used as a proxy for socioeconomic status).*<br>*Provide clear definitions of the relevant terms used, how they were provided (by the participants/respondents, the researchers, or third parties), and the method(s) used to classify people into the different categories (e.g. self-report, census or administrative data, social media data, etc.)*<br>*Please provide details about how you controlled for confounding variables in your analyses.*
Population characteristics	*Describe the covariate-relevant population characteristics of the human research participants (e.g. age, genotypic information, past and current diagnosis and treatment categories). If you filled out the behavioural & social sciences study design questions and have nothing to add here, write "See above."*
Recruitment	*Describe how participants were recruited. Outline any potential self-selection bias or other biases that may be present and how these are likely to impact results.*
Ethics oversight	*Identify the organization(s) that approved the study protocol.*

Note that full information on the approval of the study protocol must also be provided in the manuscript.

Field-specific reporting

Please select the one below that is the best fit for your research. If you are not sure, read the appropriate sections before making your selection.

- [x] Life sciences
- [ ] Behavioural & social sciences
- [ ] Ecological, evolutionary & environmental sciences

For a reference copy of the document with all sections, see nature.com/documents/nr-reporting-summary-flat.pdf

Life sciences study design

All studies must disclose on these points even when the disclosure is negative.

Sample size	The total number of AFM measurements performed was at least 8000 measurements in each condition. The total number of aggregates counted in bead and cell aggregation experiments was at least 400 aggregates for each condition. The total number for co-localization analysis was 163 for “WT-CQY” and 140 for “WT+CQY”. The total number of FRAP measurement was 20 for each condition.
Data exclusions	No data was excluded from the analysis.
Replication	Molecular dynamics simulations for each condition was replicated five times. AFM data for each condition was replicated two times. All cell-related experiments were replicated three times (biological replicates). FRAP data was replicated three times (biological replicates).
Randomization	In every AFM experiment, the cantilever would randomly sample a 40um x 40um area yielding ~2000 force-distance traces. Fluorescence imaging data was randomly selected from a 22 mm * 22 mm cover slips.
Blinding	AFM measurements were performed in a blind fashion because the AFM tip randomly pressed on a substrate without user input. AFM data was analyzed by fitting to well-defined physical models using predefined criteria (described in the methods) to minimize subjectivity. All imaging data was acquired randomly from the samples.

Reporting for specific materials, systems and methods

We require information from authors about some types of materials, experimental systems and methods used in many studies. Here, indicate whether each material, system or method listed is relevant to your study. If you are not sure if a list item applies to your research, read the appropriate section before selecting a response.

Materials & experimental systems

n/a	Involved in the study
[ ]	[x] Antibodies
[ ]	[x] Eukaryotic cell lines
[x]	[ ] Palaeontology and archaeology
[x]	[ ] Animals and other organisms
[x]	[ ] Clinical data
[x]	[ ] Dual use research of concern
[x]	[ ] Plants

Methods

n/a	Involved in the study
[x]	[ ] ChIP-seq
[x]	[ ] Flow cytometry
[x]	[ ] MRI-based neuroimaging

Antibodies

Antibodies used	Primary antibodies:<br>CQY684: produced by Genescript<br>Anti-mcherry: AB_2942805, Supplier- Invtrogen, Catalog number - PA5-143576<br>Anti-mcherry: E5D8F, Supplier- Cell Signaling Technology, Catalog number - 43590<br>Anti-p120: AB_397537, Supplier- BD Transduction Laboratories, Catalog number – 610133<br>Anti- LAMP-1: eBioH4A3, Supplier- Invtrogen, Catalog number - 14-1079-80<br><br>Secondary antibodies:<br>Goat Anti-Mouse IgG (H+L)-HRP Conjugate, Supplier- BioRad, Catalog number- 1706516<br>Goat anti-Rabbit IgG (H+L)-HRP Conjugate, Supplier- Invtrogen, Catalog number- 31460<br>Goat anti-Human IgG (H+L)-HRP Conjugate, Supplier- Invtrogen, Catalog number- 31410<br>Goat anti-Chicken IgY (H+L)-Alexa Fluor 488, Supplier- Invtrogen, Catalog number- A-11039<br>Goat anti-mouse IgY (H+L)-Alexa Fluor 594, Supplier- Invtrogen, Catalog number- A-11005
Validation	All the antibodies were purchased from commercial vendors and were validated by the manufacturer.CQY684 was validated by western blot that we performed.

Eukaryotic cell lines

Policy information about cell lines and Sex and Gender in Research

Cell line source(s)	Cells lines used were generated in-house (as described in the methods).
Authentication	Rescued cell line were verified using Western Blots.
Mycoplasma contamination	These cell lines were not tested for mycoplasma contamination.
Commonly misidentified lines<br>(See ICLAC register)	There were no commonly misidentified cell lines used in this study.
